# 3D-Printed Wearable Sensors for the Identification of Shoulder Movement Planes

**DOI:** 10.3390/s25185853

**Published:** 2025-09-19

**Authors:** Alfredo Dimo, Umile Giuseppe Longo, Pieter D’Hooghe, Alessandro de Sire, Rocco Papalia, Emiliano Schena, Daniela Lo Presti

**Affiliations:** 1 Department Traumatology and Sports Medicine, Fondazione Policlinico Universitario Campus Bio-Medico, Via Alvaro del Portillo, 00128 Rome, Italy; a.dimo@policlinicocampus.it (A.D.); r.papalia@policlinicocampus.it (R.P.); 2 Fondazione Policlinico Universitario Campus Bio-Medico, Via Alvaro del Portillo, 200, 00128 Rome, Italy; e.schena@unicampus.it (E.S.); d.lopresti@unicampus.it (D.L.P.); 3 Aspetar Orthopedic and Sports Medicine Hospital, Aspire Zone, Sportscity Street 1, Doha P.O. Box 29222, Qatar; pieter.dhooghe@aspetar.com; 4 Department of Medical and Surgical Sciences, University of Catanzaro “Magna Graecia”, 88100 Catanzaro, Italy; alessandro.desire@unicz.it; 5 Research Center on Musculoskeletal Health, MusculoSkeletalHealth@UMG, University of Catanzaro “Magna Graecia”, 88100 Catanzaro, Italy; 6 Department of Engineering, Università Campus Bio-Medico di Roma, Via Alvaro del Portillo, 00128 Rome, Italy

**Keywords:** wearable sensor, fiber Bragg grating, 3D printing, shoulder kinematics, rehabilitation monitoring, motion plane identification, additive manufacturing

## Abstract

Rotator cuff injuries are a leading cause of shoulder disability, directly impacting joint mobility and overall quality of life. Effective recovery in these patients depends not only on surgical intervention, when necessary, but also on accurate and continuous monitoring of joint movements during rehabilitation, especially across multiple anatomical planes. Traditional tools, such as clinical assessments or motion capture systems, are often subjective or expensive and impractical for routine use. In this context, wearable devices are emerging as a viable alternative, offering the ability to collect real-time, non-invasive, and repeatable data, both in clinical and home settings. This study presents innovative wearable sensors, developed through 3D printing and integrated with fiber Bragg grating technology, designed to detect the shoulder’s planes of motion (sagittal, scapular, and frontal) during flexion–extension movements. Two wearable sensors made of thermoplastic polyurethane (TPU 85A and 95A) were fabricated and subjected to metrological characterization, including strain and temperature sensitivity, hysteresis error, and tear resistance, and tested on eight healthy volunteers. The results demonstrated high discriminative ability, with sensitivity values up to 0.76 nm/mε and low hysteresis errors. The proposed system represents a promising, cost-effective, and customizable solution for motion monitoring during shoulder rehabilitation.

## 1. Introduction

Shoulder musculoskeletal disorders are a major cause of pain and disability in the adult population, with a significant impact on quality of life and the ability to perform daily and work activities [1,2]. Among these pathologies, rotator cuff (RC) lesions are among the most frequent and serious [3,4,5,6]. These lesions, of traumatic or degenerative nature, compromise the stability and mobility of the scapulohumeral joint, causing pain, reduced range of motion (ROM), loss of strength, and significant functional limitations [7,8]. When conservative therapies, such as physiotherapy or pharmacological treatment, are not sufficient, surgery is often resorted to [9]. However, the success of surgery does not depend exclusively on the surgical intervention, but also on the quality of the post-operative rehabilitation protocol, whose goal is the complete recovery of joint function. In fact, the shoulder is one of the most complex joints from a biomechanical point of view, since it involves coordinated movements between the humerus, scapula, and clavicle and has three main degrees of freedom [10]. For this reason, an effective rehabilitation program must consider the three-dimensionality of joint movement, including specific exercises in the different anatomical planes (sagittal, frontal and scapular) and aim at the progressive normalization of the ROM [11,12,13,14].

To monitor rehabilitation progress and evaluate the effectiveness of the treatment, clinical tests and motion capture systems are currently used. The former, based on direct observation and palpation by the therapist, are subject to inter-operator variability and subjective component [15]. The latter guarantee precise and objective measurements, but require structured environments, expert personnel, and high economic investments [16].

To overcome these limitations, scientific research is increasingly oriented towards the design and adoption of wearable devices, capable of acquiring data continuously, objectively and non-invasively, both in clinical and home settings [17,18]. These tools are rapidly establishing themselves as fundamental elements in the medical landscape, thanks to their ability to monitor crucial physiological parameters, such as respiratory activity and heart rate, in real time [19,20]. This possibility allows us to obtain a more detailed picture of the physiological state of the patient, effectively supporting the therapeutic path and facilitating timely interventions in case of anomalies. At the same time, wearables are able to analyze joint movements with high precision, as well as measure biomechanical and postural parameters, thus finding application in different fields such as rehabilitation, sports medicine, and injury prevention [21,22].

Among the most widespread and advanced technologies used in wearable devices, inertial measurement units (IMU), piezoresistive sensors, and smart fabrics stand out. IMUs, which combine accelerometers, gyroscopes, and magnetometers, are a valuable tool for the detailed study of body kinematics, since they allow us to acquire extremely precise data regarding the movements and orientation of the limbs and trunk [23,24]. However, these technologies have some important limitations such as the phenomenon of signal drift over time, which can alter the quality of the information collected; furthermore, calibration procedures are often complex and delicate, while incorrect sensor positioning can significantly affect the accuracy of measurements, thus compromising their overall reliability.

In a complementary way, piezoresistive sensors and conductive fabrics are characterized by their high flexibility and comfort during prolonged use, characteristics that make them particularly suitable in contexts where continuous and minimally invasive monitoring is required [25].

However, despite these advantages, these technologies are affected by hysteresis phenomena, which can compromise the fidelity of measurements, show a certain sensitivity to temperature variations, which can cause signal drift, and the repeatability of the collected data is often limited. These factors reduce their precision and reliability, representing an obstacle to their use in clinical settings where rigorous and constant measurement is essential [26].

In this context, fiber optic Bragg grating (FBG) sensors are emerging as one of the most promising solutions. These sensors have high sensitivity to mechanical deformation, light weight, immunity to electromagnetic interference, and multiplexing capabilities [27,28,29,30,31,32].

These characteristics make them particularly suitable for integration into wearable devices intended for measuring joint movement, particularly for shoulder monitoring, where precise and continuous measurement of ROM and motion planes is required [33,34].

However, the natural fragility of optical fibers requires their protection through adequate encapsulation in materials that are both flexible and resistant, such as silicones and elastomers. While ensuring effective protection, these materials require relatively long processing times and the need for special molds for their production [35,36,37]. An innovative breakthrough in this field is represented by the integration of FBG sensors within structures made through three-dimensional printing [38,39]. This approach combines the advantages of FBG technology with the potential offered by 3D printing, overcoming the typical limitations of traditional encapsulation methods. The result is greater freedom in design, significantly reduced production times, and a high degree of customization, adaptable to the specific anatomical characteristics of the patient [40,41].

Although the clinical adoption of this integrated technology is still in its early stages, it is rapidly gaining ground as one of the most promising solutions in the development of smart wearable devices for advanced healthcare applications. Preliminary research has shown that this combination can significantly improve measurement accuracy, overall mechanical resistance, and perceived user comfort, which are key aspects to ensure effective and continuous monitoring during rehabilitation treatments [42,43].

This study presents two 3D-printed wearable devices, integrated with an FBG sensor, developed to identify the shoulder’s plane of motion: sagittal, frontal, and scapular. The work is divided into four main phases:

(i) design and manufacturing of the wearable systems through 3D printing using two different polymer materials, TPU 95A and 85A;
(ii) metrological characterization of each sensor, evaluating its sensitivity to strain and temperature, and its hysteresis error;
(iii) a pull-out test to determine the strength of the interfacial bond between the optical fiber and the printed polymer structure;
(iv) a preliminary validation conducted on eight healthy subjects, aimed at verifying the system’s ability to detect shoulder joint movements and correctly distinguish the three anatomical planes of motion.

## 2. The 3D-Printed Wearable Sensors: Sensing Principle, Design, and Fabrication

### 2.1. Working Principle

The FBG sensor is an optical device that is made by inscribing a periodic modulation of the refractive index into the core of a single-mode optical fiber, through exposure to an intense UV beam. An optical fiber is composed of a core, in which the light propagates, surrounded by a cladding with a lower refractive index, which allows the light to be guided by total internal reflection [44,45]. In addition, the fiber is coated with a protective layer (coating), which helps preserve its mechanical integrity and facilitates its integration into application-specific devices. The result of the incision is a structure that selectively reflects a specific wavelength of the incident light, known as the Bragg wavelength (λ_B_), while transmitting all the other spectral components. The λ_B_ depends on the effective refractive index of the fiber core (n_eff_) and the grating pitch (Λ), according to the relationship:

λ_B_ = 2n_eff_Λ (1)

When the grating is stressed by mechanical deformations (ε) or temperature variations (ΔT), both n_eff_ and Λ change, causing a shift in the reflected wavelength (Δλ_B_). This principle makes FBGs extremely sensitive instruments for monitoring mechanical and thermal parameters. The spectral variation can be described by the following equation:

Δλ_B_ = λ_B_[(1 − pe)ε + (α + ζ)ΔT] (2)

where pe is the optical strain coefficient, α the thermal expansion coefficient, and ζ the thermo-optical coefficient [46,47,48].

In the present study, the FBG is integrated into a 3D-printed TPU structure with customized geometry, designed to ensure protection of the optical fiber and effective transmission of mechanical stresses. The proposed solution is designed to monitor shoulder movements, discriminating between the sagittal, scapular, and frontal planes during flexion–extension (F/E) exercises. During the movement, the deformation generated by the upper limb is transmitted to the sensor: at the end of the flexion, the maximum ε and therefore the maximum Δλ_B_ are recorded, while at the end of the extension, the sensor undergoes a release, corresponding to the minimum Δλ_B_.

### 2.2. Design

**Figure 1:**
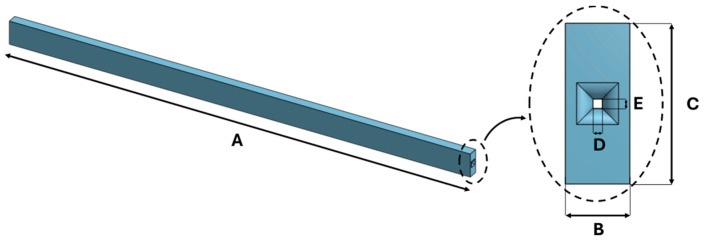
The front and lateral views of the CAD model of the 3D-printed sensor.

In this study, two flexible sensors for wearable applications were designed to identify the shoulder motion plane. The geometry, identical for both sensors, was defined through CAD modeling using OnShape^®^ software (https://www.onshape.com/en/, accessed on 20 June 2025), with a cubic shape designed to adapt to the skin and promote uniform stress distribution. During the design phase, a dedicated longitudinal channel for housing the optical fiber was included, located in the middle layer of the sensor, to ensure fiber protection during use and printing and to promote proper transmission of mechanical deformations. The design and dimensions are shown in Figure 1 and Table 1, respectively.

In this preliminary study, the sensors were designed and fabricated with identical dimensions (Table 1), in order to ensure comparability between the two TPU materials tested. Nevertheless, subject-specific sensor scaling could further improve anatomical adaptability and reduce variability in measurements, and this aspect will be considered in future developments.

### 2.3. Fabrication

The manufacturing process of the 3D printed wearable sensors is divided into three main phases:

- Pre-processing

The CAD model is imported into the Cura^®^ slicing software (version 5.4.0), where the printing parameters are defined. For this study, two sensors were fabricated with identical shape, dimensions, and integration method, but using different materials: one made of TPU 85A, which is softer and more flexible, and the other of TPU 95A, which is more rigid and structurally stable [49].

A triangular infill pattern with a density of 20% was selected for both versions to achieve a balance between structural flexibility, adaptability to shoulder movements, and adequate protection of the optical fiber [50,51]. Once the g.code file is generated, printing is initiated using the Ultimaker S5 3D printer (UltiMaker B.V., Utrecht, The Netherlands).

- Production

**Figure 2:**
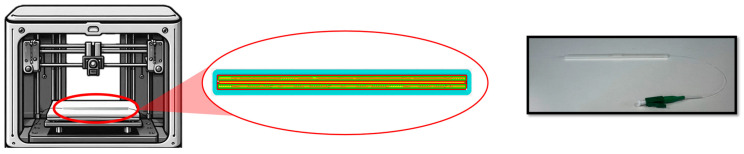
Fabrication step with a zoom on the printed layer (i.e., the 10th one) with the channel for the FBG embedment and the final prototype.

During printing, the TPU filament is extruded and deposited layer by layer. The printer is programmed to pause automatically at the tenth layer, which corresponds to the height of the central channel designed to house the optical fiber. At this stage, the fiber is manually inserted, carefully positioned, and pre-tensioned using adhesive tape to ensure stability and proper alignment. Once the fiber is in place, the print resumes until the sensor is fully completed (Figure 2). The thickness of the TPU encapsulation was selected as a suitable compromise between mechanical robustness, strain sensitivity, and user comfort, while still ensuring adequate protection of the embedded fiber.

The use of two different types of TPU was intended to evaluate how the mechanical properties of the material affect the sensor’s response.

In both sensors, a 5 mm long FBG coated in acrylate and supplied by AtGrating was embedded. The central wavelength of the grating was 1533 nm for the TPU 85A sensor and 1545 nm for the TPU 95A sensor, with reflectivity greater than 94% and 84%, respectively.

- Post-processing

After printing is complete, each sensor is carefully removed from the print bed using a spatula. This step requires particular attention to avoid applying pressure or torsional forces to the integrated FBG, which is fragile and could easily break.

## 3. Metrological Characterization

The FBG is integrated inside a polymer matrix therefore the metrological characteristics of the whole custom sensing element have to be experimentally assessed. In particular, we focused on the sensitivity of the sensor to mechanical deformation (S_ε_) and temperature (S_T_). A subsequent evaluation of hysteresis error (e_H_) was also performed at two different stress rates.

### 3.1. Response to Strain

For the characterization of the S_ε_, tensile tests were performed using a universal testing machine (Instron 3365, Norwood, MA, USA) equipped with a 500 N load cell. Both 3D-printed sensors were subjected to an axial deformation ranging from 0% to 1% of the initial length l_0_ = 50 mm, with an elongation rate of 1 mm/min, to simulate quasi-static conditions. Each test was repeated ten times for each sensor, in order to evaluate the repeatability of the response.

During the tests, the variation of the Δλ_B_ was recorded using a high-resolution optical interrogator (si255 based on HYPERION platform; Micron Optics Inc., Atlanta, GA, USA), with an acquisition frequency of 100 Hz.

All experimental data were exported and analyzed in MATLAB^®^ in order to obtain the calibration curve (Δλ_B_ vs. ε). The expanded uncertainty was estimated by assuming a Student t-distribution with nine degrees of freedom and a confidence level of 95%, calculating the product between the standard uncertainty and the coverage factor k = 2.262.

**Figure 3:**
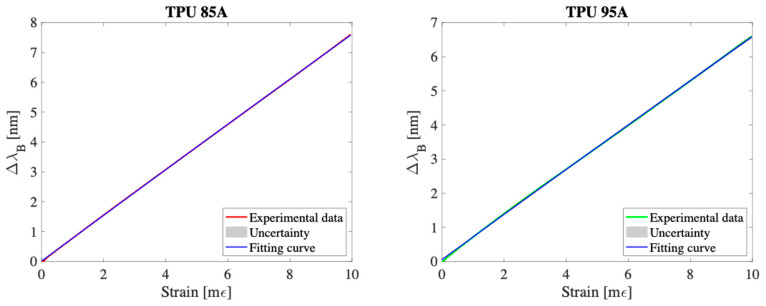
The Δλ_B_ vs. mε experimental data (red line for TPU 85A and green line for TPU 95A), the fitting curve (blue line), and the expanded uncertainty (gray shadow area).

Figure 3 reports the mean curve Δλ_B_ vs. ε, the related calibration curve and the extended uncertainty range. The calibration curve that best approximates the experimental trend is a linear curve, confirming the proportionality between the applied deformation and the variation of the reflected wavelength.

The mean sensitivity to strain, indicated as $S_{\varepsilon }^{mean}$ was calculated according to the following expression:

(3) $$S_{\varepsilon }^{mean}=\frac{{\Delta \lambda }_{B}\left(\varepsilon^{max}\right)-{\Delta \lambda }_{B}(\varepsilon^{0})}{\varepsilon^{max}-\varepsilon^{0}}$$

where Δλ_B_(ε*^max^*) represents the wavelength variation at the maximum applied strain value (1% of l_0_) and Δλ_B_(ε^0^) the initial value.

By applying Equation (3), an average sensitivity equal to 0.76 nm/mε was obtained for the TPU 85A sensor and 0.66 nm/mε for the TPU 95A sensor, highlighting a greater mechanical response of the softer material. Furthermore, the low value of the experimental uncertainty in the ten tests confirms the high repeatability of the sensors’ response to mechanical deformation (grey area in Figure 3).

### 3.2. Response to Temperature

To evaluate the S_T_ of the two sensors, they were placed inside a temperature-controlled climate chamber (BINDER™ Series KBF-S ECO Solid.Line) and exposed to a temperature variation between 20 °C and 50 °C. Temperature reference measurements were performed using a bare FBG sensor (λ_B_ = 1557 nm, length = 10 mm, acrylate coating, reflectivity > 90%, AtGrating), whose temperature sensitivity, provided by the manufacturer, is equal to ~0.01 nm/°C. Both the sensors and the reference sensor were interrogated by the optical interrogator with an acquisition rate of 100 Hz.

**Figure 4:**
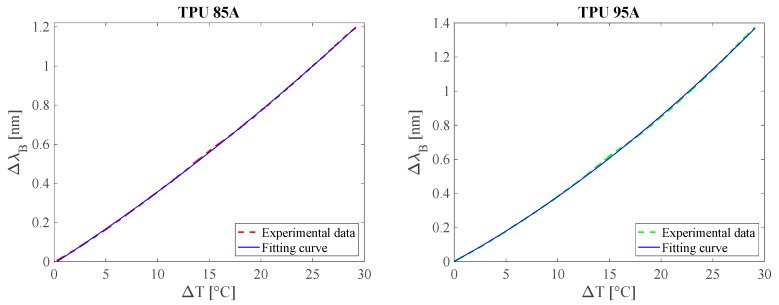
The Δλ_B_ vs. T experimental data (red dotted line for TPU 85A and green dotted line for TPU 95A) and the fitting curve (blue line).

The calibration curves Δλ_B_ vs. T, shown in Figure 4, were obtained for both sensors and approximated by second-order polynomial fitting, in order to model the non-linear behavior observed in the experimental data. To determine the mean temperature sensitivity ($S_{T}^{mean})$ the following expression was used:

(4) $$S_{T}^{mean}=\frac{{\Delta \lambda }_{B}\left(T^{max}\right)-{\Delta \lambda }_{B}\left(T^{0}\right)}{T^{max}-T^{0}}$$

where Δλ_B_(T*^max^*) and Δλ_B_(T^0^) represent the wavelength variation at the end and beginning of the temperature interval (T*^max^* = 50 °C, T^0^ = 20 °C), respectively.

The analysis showed that the sensor encapsulated in TPU 85A has an average thermal sensitivity of 0.041 nm/°C, while the one in TPU 95A shows a slightly higher value of 0.047 nm/°C. This difference can be attributed to the different mechanical and thermal expansion properties of the polymer materials used. In particular, the higher stiffness of TPU 95A generates a more marked thermal stress on the optical sensor, contributing to the increase in the response in terms of Δλ_B_. This increase in thermal sensitivity can primarily be attributed to the higher stiffness of TPU 95A, which leads to a more effective transfer of thermally induced stress to the fiber. While both TPU grades have similar coefficients of thermal expansion (CTE ≈ 160–200 × 10^−6^ /°C), the stiffer TPU 95A constrains the fiber more during thermal expansion, resulting in greater axial strain [52].

### 3.3. Hysteresis Cycle

**Figure 5:**
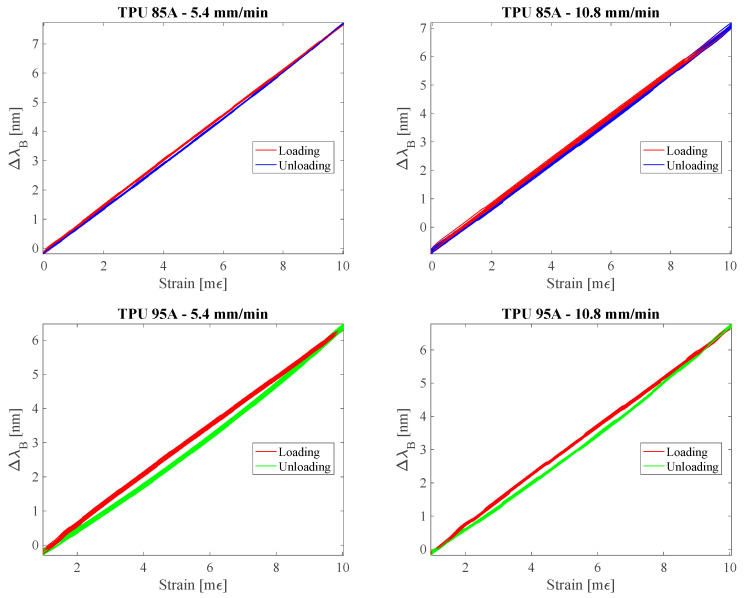
Loading (red lines) and unloading (blue lines for TPU 85A and green lines for TPU 85A) phases of the ten hysteresis cycles at different speeds (5.4 mm/min on the left and 10.8 mm/min on the right).

For the evaluation of the e_H_, the same experimental setup employed for the estimation of the Sε was used. The analysis was conducted through a dynamic test, during which each sensor was subjected to ten consecutive loading and unloading cycles. In each cycle, the sensor was progressively stretched from ~0% to 1% of its initial length (l_0_) and subsequently released from 1% to ~0%. The test was performed at two different stretching speeds: 5.4 mm/min and 10.8 mm/min (Figure 5). These speeds were selected to represent the extremely controlled and gradual motion typical of early-phase shoulder rehabilitation protocols, particularly following RC tear surgery.

During the whole experiment, data from both the encapsulated sensors and the tensile machine were acquired with a sampling frequency of 100 Hz. The collected data were processed in MATLAB^®^ environment to calculate the percentage hysteresis error (e_H_%), according to the following expression:

(5) $$e_{H}\%=\frac{e_{max}}{max({\Delta \lambda }_{B}^{loading})}\cdot 100$$

where emax represents the maximum difference of Δλ_B_ between the loading and unloading phases for the same strain value, and max(${\Delta \lambda }_{B}^{loading}$) represents the maximum response value during the loading phase.

The results highlighted a differentiated behavior between the two polymeric materials. The TPU 85A sensor showed a hysteresis error of 2.84% and 3.92% for the velocities of 5.4 mm/min and 10.8 mm/min, respectively. In contrast, the TPU 95A sensor showed significantly higher values, equal to 6.76% and 6.61% for the same velocities.

These differences can be attributed to the viscoelastic characteristics of the two materials. In particular, the softer behavior of TPU 85A seems to reduce the energy dissipation phenomenon during the loading-unloading cycles, while the higher stiffness of TPU 95A may increase the viscoelastic effects, contributing to a more marked hysteresis error. Nevertheless, the values obtained fall within a range compatible with the typical velocities of the joint movement during the post-operative rehabilitation phase of the RC.

### 3.4. Pull-Out Test

In order to evaluate the resistance of the interface between the optical fiber and the 3D printed polymer matrix, a pull-out test was conducted on samples made of TPU 85A and TPU 95A. In particular, for each material, three sensors were prepared identical in geometry to those used in the functional tests, with the only difference that, instead of the FBG sensor, a standard optical fiber (external diameter 250 μm, not sensorized) was integrated.

The tests were performed using a universal testing machine (Instron 3365, Norwood, MA, USA), equipped with a 500 N load cell. Each sample was subjected to a traction at a constant speed of 5 mm/min, with a maximum stroke of 10 mm. The test setup involved locking the sensor body between the lower jaws and fixing the optical fiber between the upper jaws. In this way, the applied load acted directly on the fiber–structure interface, simulating the most critical mechanical conditions for adhesion.

The results obtained showed that, for all the tested samples, the maximum applicable force before the functional loss was always higher than 12 N.

**Figure 6:**
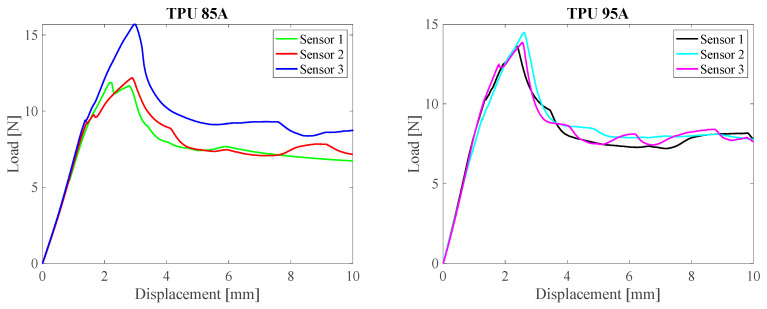
Representative force–displacement curves obtained from the pull-out test. The curve on the left corresponds to the sensor fabricated with TPU 85A, while the one on the right refers to the sensor made with TPU 95A.

In none of the tested samples was the complete detachment of the optical fiber from the 3D printed structure observed, nor were there any filament breaks. As highlighted in Figure 6, when a certain force threshold was exceeded, a relative slippage phenomenon occurred between the coating and the cladding of the fiber, without the fiber being unmoored from the polymer matrix.

These results highlight a high mechanical cohesion at the fiber–matrix interface, both in TPU 85A and TPU 95A samples, demonstrating the effectiveness of the integration process adopted.

## 4. Preliminary Evaluation of the Wearable Sensor Capability of Monitoring Shoulder Movements

### 4.1. Experimental Setup and Protocol

To explore the sensors’ ability to recognize the shoulder motion plane, a preliminary study was conducted on a group of eight healthy volunteers (Table 2), free from upper limb musculoskeletal problems.

The study was conducted in compliance with the ethical principles of the Declaration of Helsinki and was approved by the Ethics Committee of the Campus Bio-Medico University of Rome (protocol code: 72.25 CET2cbm ATLETIC, date of approval: 27 May 2025). All subjects read the protocol and signed an informed consent before participating.

Each volunteer was tested using two different wearable sensors. For each sensor, three separate acquisitions were performed, each one separated by the removal and subsequent manual reapplication of the device. This procedure allowed to evaluate the replicability of the system in real conditions of use, considering that the positioning of the sensor is done manually and can represent a potential source of variability.

**Figure 7:**
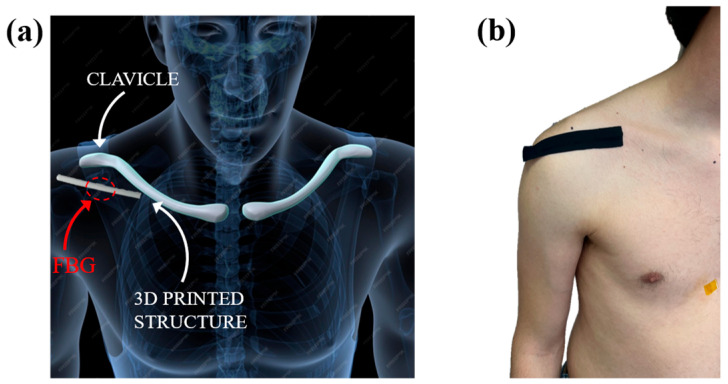
Positioning of the device in the antero-lateral region of the shoulder: (**a**) Schematic representation of the sensor placement; (**b**) Wearable device attached to the subject.

The device was applied directly to the skin in the antero-lateral region of the shoulder, immediately below the clavicle and close to the acromial end, as illustrated in Figure 7. This specific anatomical area was selected because it represents a region that undergoes significant deformations during upper limb movements in all the main planes. Furthermore, the absence of superficial bony prominences and the presence of underlying soft tissue favor a good mechanical transmission of deformations to the sensor, improving the quality of the collected signal [53,54].

To ensure correct adherence of the sensor to the skin surface during the entire test, an elastic adhesive tape (Alpidex^®^) was used, applied in such a way as to ensure mechanical stability without limiting movements.

**Figure 8:**
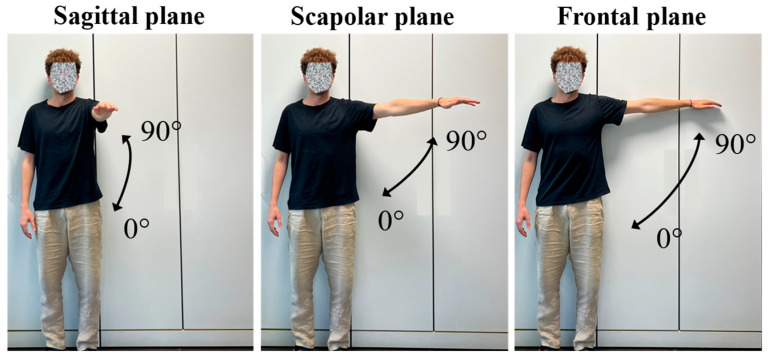
Sequence of flexion–extension movements of the upper limb in the three anatomical planes: sagittal, scapular, and frontal.

During the experiment, participants were asked to perform a F/E movement of the upper limb in three different anatomical planes: sagittal, scapular and frontal. Each sequence consisted of an extension from 0° to 90°, followed by a return to the starting position, with a pause of approximately 3 s between each execution (Figure 8). The movements were performed at free speed, in order to simulate a natural physiotherapy execution, without imposed constraints, as can be seen in the Supplementary Material Video S1.

### 4.2. Results

**Figure 9:**
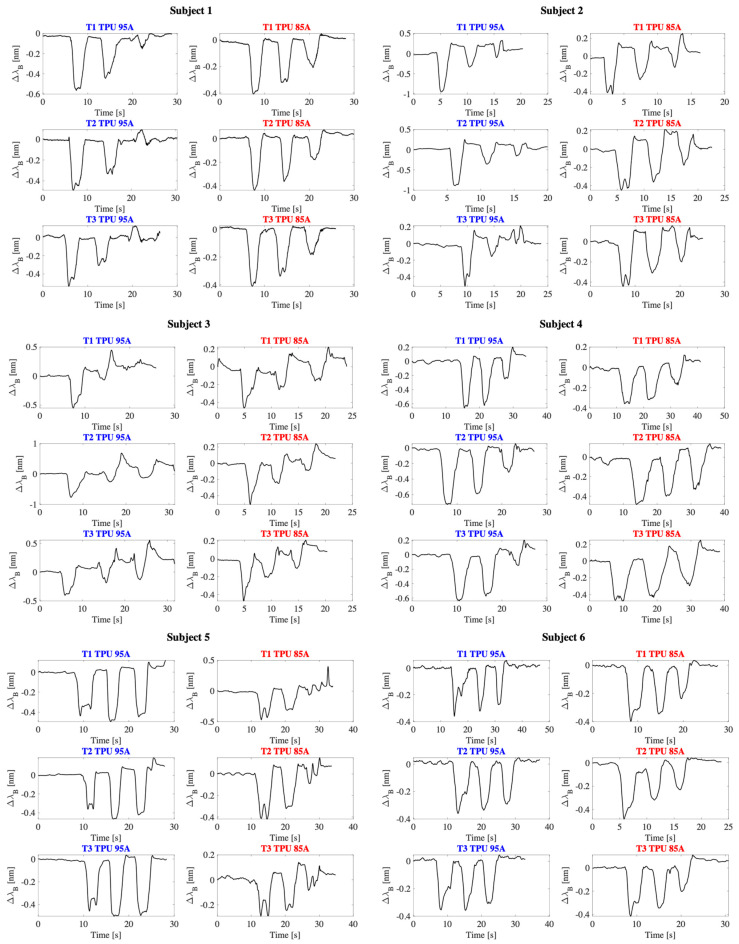

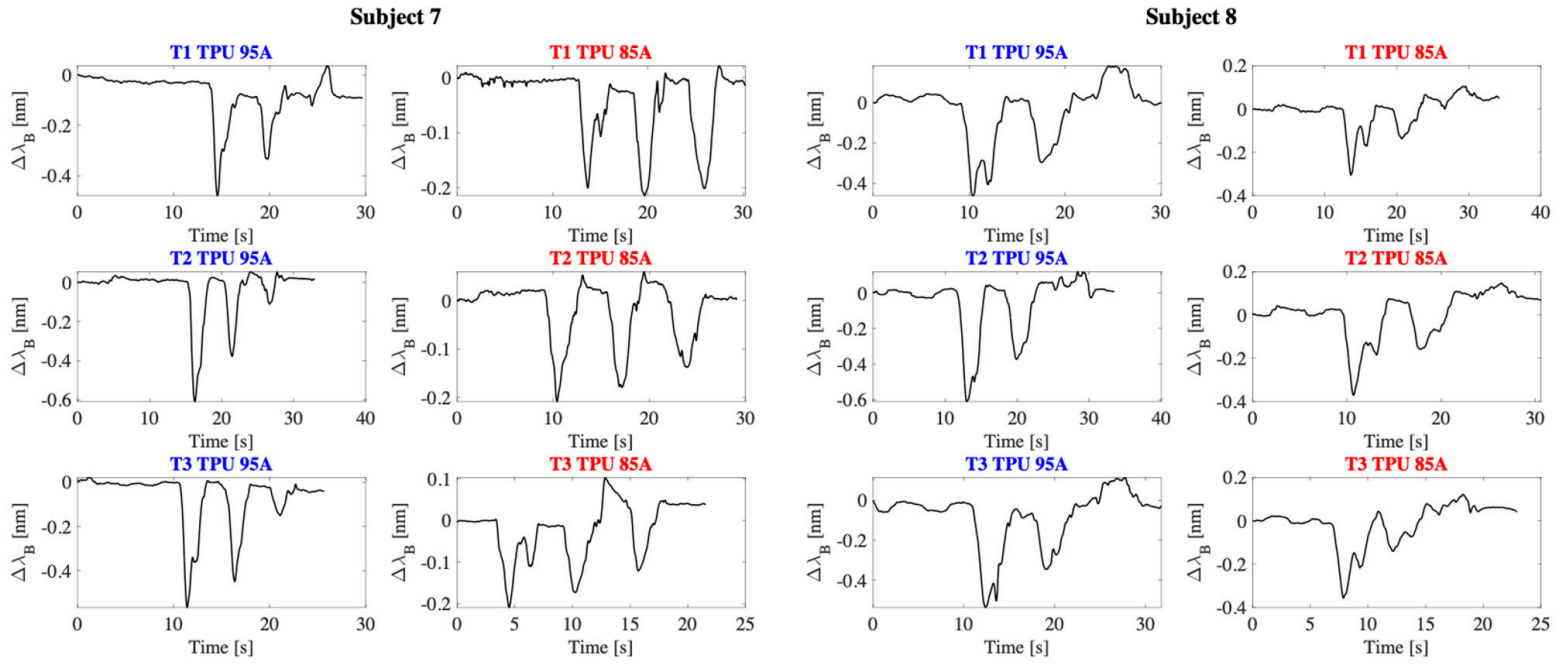
Wearable sensor output for both materials (TPU 85A and 95A), considering the three trials (T1, T2 and T3) conducted on each of the 8 subjects.

The data acquired by the 3D printed sensor were recorded at a sampling rate of 100 Hz using the optical interrogator and subsequently analyzed in MATLAB^®^ environment (version R2020b). For each task performed by the subjects, the signal was segmented with the aim of identifying the output variations associated with the F/E movements in the three anatomical planes (Figure 9).

The data analysis highlighted the capability of both wearable sensors to discriminate the three motion planes, confirming the effectiveness of the system in detecting shoulder dynamics. In particular, the Δλ_B_ signal over time showed a variation consistent with the F/E movements of the arm.

**Figure 10:**
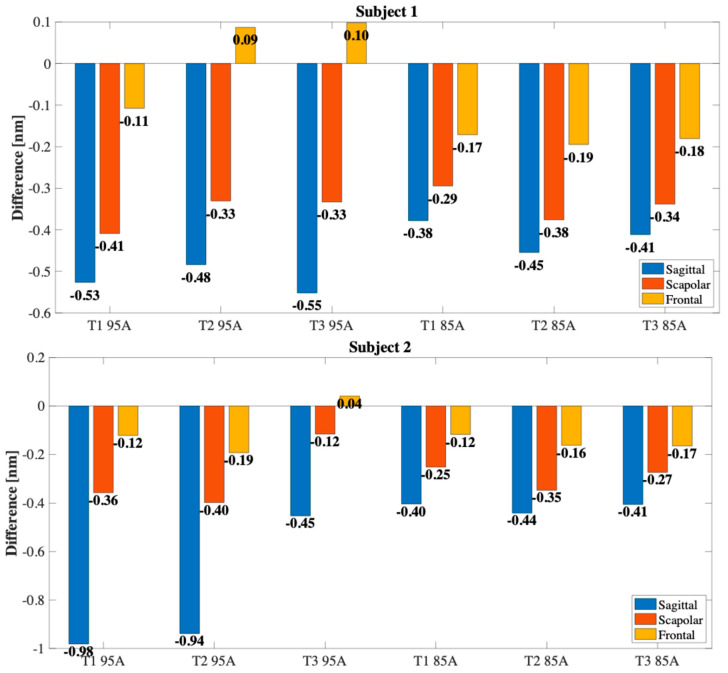

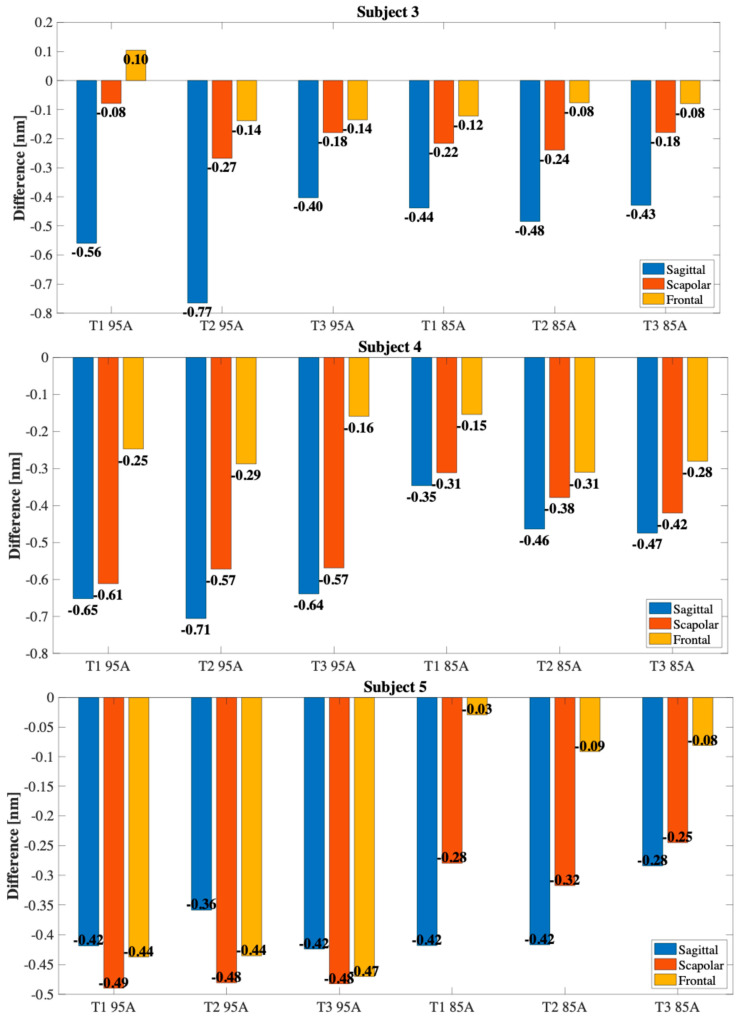

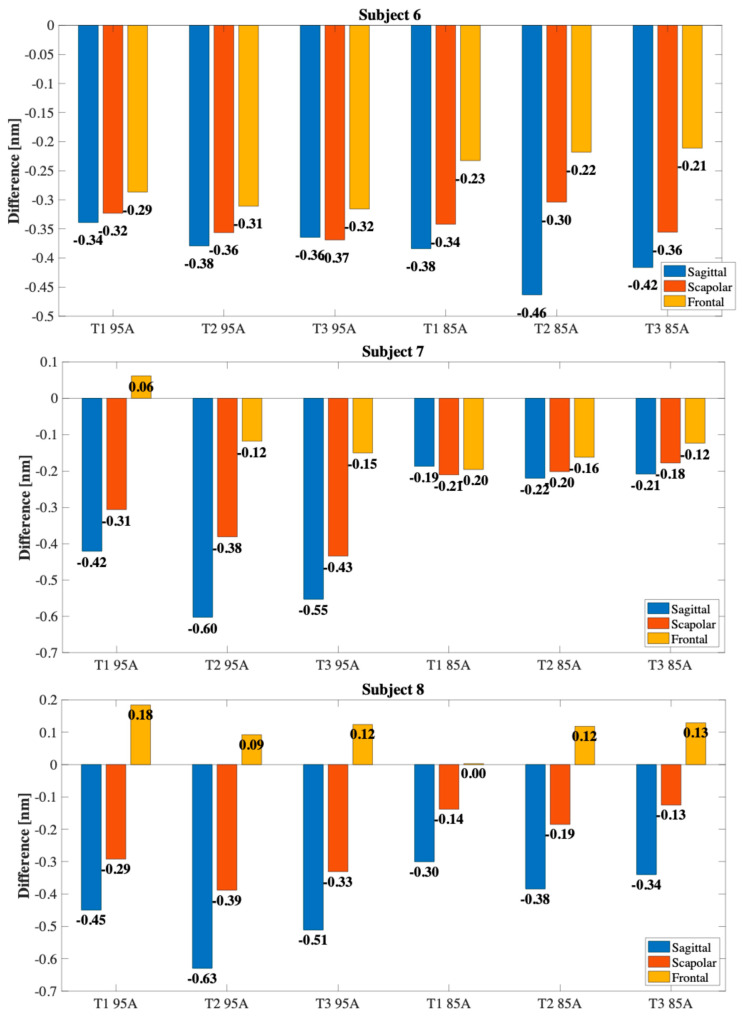
Overall results of Δλ_B_ variations by subject and motion plane, distinguished by sensor material (TPU 95A and TPU 85A) and trial number (T1, T2 and T3).

The overall results are reported in Figure 10, where the histograms with the Δλ_B_ spectral variations detected for each motion plane and for each subject are illustrated. The observed interindividual differences can be attributed to morphological characteristics, differences in individual biomechanics, or variations in the way the motor task is performed. Similar to what was observed in previous studies, the sensor response was more pronounced in the sagittal plane, where F/E movements induce larger rotations of the scapula and significant translations of the clavicle. On the contrary, compression was less marked in the frontal plane, while an intermediate response was detected in the scapular plane, compatible with the combined nature of the movement.

In addition, in some trials a traction response, rather than a compression one, was observed exclusively in the frontal plane (as observed, for example, in the first trial with the TPU 95A sensor in subject 3 and in the first trial with the TPU 85A sensor in subject 5). This behavior, although less frequent, can be attributed both to slight variations in the geometry of the sensor positioning and to the specific nature of the movement in this plane, where, depending on the orientation of the arm and the morphology of the subject, it is possible that sensor elongation forces, rather than compression, are generated. This effect highlights how the direction of the deformation is not always univocal and can be influenced by individual biomechanical and anatomical factors.

Although the TPU 95A sensor showed lower sensitivity and higher error in vitro compared to TPU 85A, its stiffer structure provided greater mechanical stability when worn, resulting in improved discrimination of shoulder movement planes during on-body tests.

Only under four conditions was it not possible to clearly discriminate all three planes of movement: for subject 7 when using TPU 85A sensor (−0.19 nm in the sagittal plane vs. −0.21 nm in the scapular plane in T1), for subject 5 when using TPU 95A sensor (−0.42 nm sagittal vs. −0.49 nm scapular in T1 and −0.36 nm sagittal vs. −0.48 nm scapular in T2), and for subject 6 when using TPU 95A sensor in T3 (−0.36 nm sagittal vs. −0.37 nm scapular). In these cases, the signal was less defined and the separation between the Δλ_B_ signal associated with the different planes was not sufficiently marked. It is likely that this limitation is attributable to specific anthropometric characteristics such as, for example, the shape of the shoulder and, above all, to the variability introduced by the positioning of the sensor, which represents a critical factor for the reliability of the measurement.

## 5. Discussion and Conclusions

This study presented two innovative wearable sensors for the estimation of the shoulder motion plane during F/E activities, based on a FBG sensor integrated into a 3D-printed polymer matrix. Compared to previous devices in the literature [46,47,48], the system stands out for its ability to discriminate between sagittal, frontal and scapular planes, a fundamental function in the rehabilitation field, especially in patients affected by RC lesions.

The use of 3D printing has allowed the creation of a compact system, easily replicable and customizable according to the patient’s anatomy. Two structurally identical sensors were printed using materials with different hardness, namely TPU 85A and TPU 95A. The sensor made of TPU 85A showed a higher sensitivity to deformation (0.76 nm/mε) compared to the one made of TPU 95A (0.66 nm/mε), thanks to the greater deformability of the material. The TPU 85A sensor showed a hysteresis error of 2.84% at 5.4 mm/min and 3.92% at 10.8 mm/min, while the TPU 95A sensor showed values of 6.76% and 6.61%, respectively. In terms of thermal response, a sensitivity of 0.041 nm/°C was observed for the TPU 85A sensor and 0.047 nm/°C for the TPU 95A sensor, higher values than a non-encapsulated FBG (~0.01 nm/°C). However, in the intended application, the temperature variations are limited and the effect can be considered negligible compared to the mechanical response of the system.

This work stands out for its originality: to our knowledge, it is the first time that 3D printing and FBG technology are combined to discriminate between different joint motion planes. Other works in the literature have explored FBG 3D printing for joint monitoring, but with different purposes. For example, Cheng-Yu et al. [55] developed a PLA ring system for elbow and knee F/E motion monitoring. Although the device was effective for angular excursions up to 90°, the use of PLA, a rigid material, limits the anatomical adaptability to the shoulder, which is the most complex and mobile joint in the human body. In contrast, our devices, made of TPU, ensure high conformability and adaptability to different anthropometric characteristics of patients. Another relevant comparison can be made with Jin et al. [56] who developed a “sensing shirt” based on textile capacitive sensors for the estimation of shoulder kinematics in three degrees of freedom. Although this system shows excellent performance in terms of accuracy (RMSE < 4.5°) and continuous tracking, it requires the use of eight sensors, dedicated electronics and a more complex manufacturing process. Our approach, on the contrary, is based on a single sensor and local variations of mechanical deformation, reducing the complexity of the system while maintaining a clinically relevant functionality, although currently limited to plane discrimination.

In addition, Dimo et al. [48] proposed a multiparametric, 3D-printed, FBG-based wearable system for monitoring shoulder movement and respiratory rate. While this device showed a sensitivity to strain of 0.65 nm/mε, a thermal sensitivity of 0.038 nm/°C, and a hysteresis error of 4.1%, our current solution demonstrates slightly higher strain sensitivity and lower hysteresis, thus improving metrological robustness. Similarly, Carnevale et al. [54] investigated a piezoresistive textile-based system for shoulder motion monitoring. Despite its good wearability, the sensor exhibited a non-linear response and hysteresis errors exceeding 10%, which limit its reliability in clinical scenarios when compared to our FBG-based approach.

In addition to the qualitative comparison, a quantitative analysis with existing technologies is reported below. Compared to other wearable sensing systems, the proposed devices show promising metrological performances. In particular, the sensitivity to strain (0.66–0.76 nm/mε) is consistent with other FBG-based approaches and higher than values typically reported for textile piezoresistive sensors, which often show non-linear responses and limited repeatability. The hysteresis error (2.84–6.76%) is lower than values usually observed in resistive or capacitive sensors (>10%) and within a clinically acceptable range for shoulder rehabilitation monitoring. Furthermore, in contrast to inertial measurement units, which suffer from drift and require continuous recalibration, our single-sensor approach does not require complex algorithms for motion plane discrimination. Finally, the reduced system complexity compared to multi-sensor smart garments (e.g., sensing shirts requiring up to eight electrodes) highlights the potential of our solution as a simple and cost-effective alternative. These aspects confirm the suitability of the proposed sensor as a competitive alternative in rehabilitation monitoring.

Although the system demonstrated reliable discrimination of sagittal, scapular, and frontal movements, this study did not include a direct quantification of the angular deviation from the ideal sagittal plane. This limitation is due to the absence of a reference motion capture system or goniometers, which prevented us from expressing the measurement error in degrees. Nevertheless, the obtained results confirm that, even without a direct estimation of the spatial angular error, the proposed sensor effectively fulfills its primary task of reliably identifying the different shoulder movement planes. Recognizing the importance of this aspect, the estimation of spatial angular error will represent a key step for future validation studies, where the proposed sensor will be benchmarked against certified optical or inertial systems and goniometers.

For completeness, Table 3 reports a summary comparison with the aforementioned studies.

To conclude, this work opens new perspectives for the development of wearable sensors, suitable for joint motion monitoring in rehabilitation. The obtained metrological performances and the ability to discriminate the shoulder motion planes are promising and represent a first step towards the integration of such devices in clinical practice. However, some critical issues remain that require further investigation. In particular, the poor repeatability between tests, attributable to variations in sensor positioning or interaction with the body, suggests the need for an individual pre-calibration phase before use. These aspects are closely related to inter-subject anthropometric variability, suggesting that a subject-specific calibration procedure could further improve measurement reliability. In addition, the use of identical sensor dimensions for all subjects, while ensuring comparability between materials, may have limited anatomical adaptability and contributed to inter-individual variability. Furthermore, to increase the automation and robustness of the system, a machine learning algorithm could be implemented to automatically identify the motion plane based on the recorded deformation pattern. Finally, future developments will include integration with additional sensory modules in order to evaluate for example also the ROM, the use of a bare FBG for thermal compensation, miniaturization of the interrogation system and clinical validation on a larger number of subjects. Future developments will also be aimed at evaluating possible design modifications (e.g., reducing the size of the sensor) and at extending the analysis to other TPU grades with larger stiffness differences, such as by comparing TPU 60A and 95A, in order to more thoroughly investigate the impact of material mechanical properties on sensor performance. In future studies, the recruitment will also be extended to female participants, in order to investigate potential sex-related differences in sensor performance and applicability.

## Figures and Tables

**Table 1:** The 3D-printed sensor dimension.

Dimension [mm]	
A	100
B	3
C	5
D	0.3
E	0.3

**Table 2:** Anthropometric characteristics of the volunteers.

Subjects	Gender	Age (y.o.) *	Height (cm) *	Weight (kg) *
S1 *	M *	24	180	78
S2	M	24	174	72
S3	M	29	186	90
S4	M	26	178	69
S5	M	26	177	80
S6	M	25	179	70
S7	M	24	175	69
S8	M	26	186	80

* S = Subject; M = Male; y.o. = years old; cm = centimeters; kg = kilograms.

**Table 3:** Summary of wearable sensing systems and their main metrological characteristics.

Author (Year)	Sensor Type	Strain Sensitivity	Temperature Sensitivity	Hysteresis Error
Dimo et al., 2024 [48]	FBG + 3D-printed multiparametric	0.65 nm/mε	0.038 nm/°C	4.1%
Carnevale et al., 2021 [54]	Piezoresistive textile	n.a. (relative resistance)	n.a	>10%
Cheng-Yu et al., 2021 [55]	FBG + 3D-printed ring (FDM)	0.42 nm/mε	n.a	5%
Jin et al., 2020 [56]	Capacitive textile (sensing shirt)	n.a	n.a	n.a. (RMSE < 4.5° for kinematics)

## Data Availability

The original contributions presented in this study are included in the article/Supplementary Material. Further inquiries can be directed to the corresponding author.

## Supplementary Materials

The following supporting information can be downloaded at: https://www.mdpi.com/article/10.3390/s25185853/s1, Video S1: manuscript-supplementary.
